# Hybrid CUSUM Change Point Test for Time Series with Time-Varying Volatilities Based on Support Vector Regression

**DOI:** 10.3390/e22050578

**Published:** 2020-05-20

**Authors:** Sangyeol Lee, Chang Kyeom Kim, Sangjo Lee

**Affiliations:** Department of Statistics, Seoul National University, Seoul 08826, Korea; ckdk95@snu.ac.kr (C.K.K.); lscho13@snu.ac.kr (S.L.)

**Keywords:** GARCH time series, change point detection, CUSUM of squares test, support vector regression, machine learning

## Abstract

This study considers the problem of detecting a change in the conditional variance of time series with time-varying volatilities based on the cumulative sum (CUSUM) of squares test using the residuals from support vector regression (SVR)-generalized autoregressive conditional heteroscedastic (GARCH) models. To compute the residuals, we first fit SVR-GARCH models with different tuning parameters utilizing a time series of training set. We then obtain the best SVR-GARCH model with the optimal tuning parameters via a time series of the validation set. Subsequently, based on the selected model, we obtain the residuals, as well as the estimates of the conditional volatility and employ these to construct the residual CUSUM of squares test. We conduct Monte Carlo simulation experiments to illustrate its validity with various linear and nonlinear GARCH models. A real data analysis with the S&P 500 index, Korea Composite Stock Price Index (KOSPI), and Korean won/U.S. dollar (KRW/USD) exchange rate datasets is provided to exhibit its scope of application.

## 1. Introduction

In this study, we aim to develop a method to detect a significant change in the conditional variance of time series with time-varying volatility using the cumulative sum (CUSUM) of squares test based on the support vector regression (SVR)-generalized autoregressive conditional heteroscedastic (GARCH) residuals. In this task, obtaining accurate predictions of volatilities is crucial because the residuals are obtained as the ratios of the observations and the forecasts of volatility. Traditionally, the prediction has been regarded as an important and challenging task in financial time series analysis owing to its non-stationary and nonlinear nature and, in particular, its volatility. The GARCH model, proposed by Engle [1] and Bollerslev [2], is the most popular model for measuring the volatility of financial time series. This model has advantages for characterizing the properties of time series well, such as persistency, heavy tails, and volatility clustering. Subsequently, several GARCH variants have been developed to better capture these properties, e.g., the asymmetric GARCH model proposed by Engle [3], the exponential GARCH (EGARCH) model proposed by Nelson [4], the threshold GARCH (TGARCH) model proposed by Zakoïan [5], the GJR-GARCH model proposed by Glosten, Jagannathan and Runkle [6], and the asymmetric power ARCH (APARCH) model proposed by Ding, Granger and Engle [7]. Refer to Carrasco and Chen [8] for the properties of these models.

Although these GARCH models perform well in general, empirical studies show that they have low forecasting performance in the presence of model misspecification. As the underlying model of time series is often difficult to identify, new analytical tools such as a neural networks (NN) and support vector machines (SVM) have been adopted as a replacement of the classical GARCH models for forecasting time series volatilities, owing to their excellent capability of approximating nonlinearity without knowing the underlying dynamic structure of time series a priori. Refer to Fernandez-Rodriguez, Gonzalez-Martel and Sosvilla-Rivero [9], Cao and Tay [10], Pérez-Cruz, Afonso-Rodriguez and Giner [11], Cherkassky and Ma [12], Chen, Härdle and Jeong [13], Shim, Kim, Lee and Hwang [14], Shim, Hwang and Seok [15], Bezerra and Albuquerque [16], and the papers cited therein. In this study, the SVM method is adopted because it is subordinate to the “structural risk minimization principle” (Vapnik [17]) and therefore seeks a balance between the model complexity and the empirical risk (Smola and Schölkopf [18]) and performs properly for relatively small datasets. In contrast, as mentioned in Tay and Cao [19] and Chen, Härdle and Jeong [13], the NN might suffer from a number of tuning parameters, difficulty in obtaining a global solution, and over-fitting.

Since Page [20], the parameter change detection problem has been a crucial issue in economics, engineering, and medicine, and numerous articles have been published in various research areas. As financial time series often suffer from structural changes owing to changes in governmental policy and critical social events and ignoring them leads to a false conclusion, the change point test has been viewed as a core research topic in time series analysis. See Csörgő and Horváth [21] and Chenand and Gupta [22] for a general review. In particular, detecting the volatility change is important in risk management since it affects the calculation of risk measurements such as value-at-risk (VaR) and expected shortfall (ES) (Kim and Lee [23]). The CUSUM test has long been used as a functional tool for detecting a change point owing to its handiness in practice. For earlier works, we refer to Inclán and Tiao [24], Kim, Cho and Lee [25], Lee, Ha, Na and Na [26], Berkes, Horvath and Kokoszka [27], Hillebrand [28], Gombay [29], and Tahmasbi and Rezaei [30]. Subsequently, numerous articles have appeared in the literature. For further developments, we refer to Ross [31], Oh and Lee [32], and the papers cited therein.

Among the CUSUM tests, the estimate-based CUSUM test has been a conventional method for detecting a change point (Lee, Ha, Na and Na [26]) for decades because it directly compares the discrepancy among sequentially obtained estimators and, as such, is quite appealing to the intuition of practitioners. This method performs well in general, but often suffers from severe instability and produces low powers, particularly when the underlying model is a GARCH type. See Kang and Lee [33] and Oh and Lee [34]. To rectify this problem, the residual-based CUSUM test has been proposed because the residuals can effectively discard the correlations of time series and highly enhance the performance of the CUSUM test in terms of stability and power. This method has been firmly advocated by Lee, Tokutsu and Maekawa [35], De Pooter and Van Dijk [36], and Lee and Lee [37]. Oh and Lee [32] recently proposed a modified residual-based CUSUM test to cope with more general location-scale time series models including GARCH models. Despite its merits, the method relies on presumed parametric models and bears the potential risk of leading to a false conclusion when the assumed models are misspecified. To resolve this problem, Lee, Lee and Moon [38] recently designated a hybrid method combining the SVR and CUSUM methods in autoregressive and moving average (ARMA) models and demonstrated its superiority over the classical ARMA-based CUSUM test when the time series has significant nonlinear characteristics. However, as the SVR-ARMA models cannot capture volatility in financial time series and the CUSUM test based on them is highly affected by volatility and easily misidentifies the change point (Lee, Lee and Moon [38]), we aim to develop a CUSUM test based on the SVR-GARCH models.

The rest of this paper is organized as follows. Section 2 introduces the SVR-GARCH model for forecasting volatilities and describes the SVR method in a general framework. Section 3 presents the principle of the CUSUM of squares test for the GARCH models and explains how to apply the SVR-GARCH method for constructing the CUSUM of squares test. Section 4 conducts Monte Carlo simulations to evaluate the performance of the proposed method. Section 5 performs a real data analysis using the S&P 500 index, KOSPI, and KRW/USD exchange rate datasets. Finally, Section 6 provides the concluding remarks.

## 2. Support Vector Regression for the GARCH Model

Let us consider the GARCH model:

(1) $$\begin{array}{cc} y_{t} & =\sigma_{t}\epsilon_{t},\\ \sigma_{t}^{2} & =g(y_{t-1}^{2},\ldots ,y_{t-p}^{2},\sigma_{t-1}^{2},\ldots ,\sigma_{t-q}^{2}), \end{array}$$

where *g* is an unknown function, p,q are nonnegative integers, and $\left\{\epsilon_{t}\right\}$ is a sequence of independent and identically distributed (i.i.d.) random variables with zero mean and unit variance. When *g* is linear, the inferences for stationary GARCH(p,q) models are well developed in the literature (Francq and Zakoïan [39] and Francq and Zakoïan [40]). Ideally, this approach provides an excellent analytic tool for prediction, but the accuracy of prediction can be deteriorated owing to a violation of the assumptions such as stationarity and linearity. As such, in this study, we use the SVR-GARCH method for prediction.

SVR is a revision of the SVM, initially proposed by Cortes and Vapnik [41], which seeks an optimal hyperplane that separates the inputs by maximizing the margins between the support vectors and the hyperplane. Equipped with a high generalization ability (Abe [42]), a notable strength of the SVM is that its usage can be extended to nonlinear prediction and classification methods because of its ability to map the inputs to a higher dimensional feature space via nonlinear kernel functions (Cortes and Vapnik [41]).

SVR aims to identify a nonlinear function of the form:

$$f\left(x\right)=w^{T}\phi \left(x\right)+b,$$

where x denotes a vector of inputs, *w* and *b* are vectors of the regression parameter, and ϕ is a known kernel function. In the context of this paper, x is comprised of squared inputs up to lag *p* ($y_{t-1}^{2},\ldots ,y_{t-p}^{2}$) and conditional variance up to lag *q* ($\sigma_{t-1}^{2},\ldots ,\sigma_{t-q}^{2}$). Notice that *w* and *b* have the same dimension as x. The optimal *w* and *b* that yield the best approximation of *f* are determined by exploiting the structure of the ϵ-insensitive loss function (Vapnik [17]):

(2) $$\begin{array}{c} L_{\epsilon }\left(y,f\left(x\right)\right)=\left\{\begin{array}{cc} |y-f(x)|-\epsilon & \mathrm{if}\;|y-f(x)|\geq \epsilon \\ 0 & \mathrm{otherwise}, \end{array}\right. \end{array}$$

which neglects the errors lying inside the ϵ-band surrounding the estimated function *f*. The SVR aims to seek a function that keeps the data inside such a band and dismisses excessive complexity (Smola and Schölkopf [18]).

Given the input vectors $x_{i}$, scalar output $y_{i}$, i=1,…,n, and a constant C>0, we construct the objective function of the SVR using the ϵ-insensitive loss function (Vapnik [17]):

(3) $$\begin{array}{c} \mathrm{minimize}\;\frac{1}{2}{\left||w|\right|}^{2}+C\sum_{i=1}^{n}\left(\xi_{1,i}+\xi_{2,i}\right), \end{array}$$

$$\mathrm{subject}\;\mathrm{to}\;\left\{\begin{array}{c} y_{i}-w^{T}\phi \left(x_{i}\right)-b\leq \epsilon +\xi_{2,i}\\ w^{T}\phi \left(x_{i}\right)+b-y_{i}\leq \epsilon +\xi_{1,i}\\ \xi_{1,i}\geq 0,\;\xi_{2,i}\geq 0, \end{array}\right.$$

where $\xi_{1,i},\xi_{2,i}>0$ denote slack variables that allow some points to lie outside the ϵ-band with a penalty and *C* denotes a trade-off between the function complexity and the training error. Notice that the former two constraints designate the upper and lower bound, respectively.

To obtain the optimal *w* and *b*, we formulate an unconstrained optimization problem using Lagrange multipliers (Smola and Schölkopf [18]):

(4) $$\begin{array}{cc} \mathrm{minimize}\;L & :=\frac{1}{2}{\left||w|\right|}^{2}+C\sum_{i=1}^{n}\left(\xi_{1,i}+\xi_{2,i}\right)\\ \; & -\sum_{i=1}^{n}\left(\beta_{1,i}\xi_{1,i}+\beta_{2,i}\xi_{2,i}\right)\\ \; & -\sum_{i=1}^{n}\alpha_{1,i}\left(\epsilon +\xi_{1,i}-y_{i}+w^{T}\phi \left(x_{i}\right)+b\right)\\ \; & -\sum_{i=1}^{n}\alpha_{2,i}\left(\epsilon +\xi_{2,i}+y_{i}-w^{T}\phi \left(x_{i}\right)-b\right). \end{array}$$

Then, the optimal solution must satisfy the following Karush–Kuhn–Tucker conditions (Vapnik [17]):

(5) $$\left\{\begin{array}{ll} w-\sum_{i=1}^{n}\left(\alpha_{1,i}-\alpha_{2,i}\right)\phi \left(x_{i}\right)=0 & \\ \sum_{i=1}^{n}\left(\alpha_{1,i}-\alpha_{2,i}\right)=0 & \\ C-\alpha_{1,i}-\beta_{1,i}=0\;\; & \forall i=1,\ldots ,n\\ C-\alpha_{2,i}-\beta_{2,i}=0\;\; & \forall i=1,\ldots ,n\\ \alpha_{1,i}\geq 0,\alpha_{2,i}\geq 0,\xi_{1,i}\geq 0,\xi_{2,i}\geq 0 & \forall i=1,\ldots ,n, \end{array}\right.$$

and:

(6) $$\left\{\begin{array}{ll} \alpha_{1,i}\left(\epsilon +\xi_{1,i}-y_{i}+w^{T}\phi \left(x_{i}\right)+b\right)=0\;\; & \forall i=1,\ldots ,n\\ \alpha_{2,i}\left(\epsilon +\xi_{2,i}+y_{i}-w^{T}\phi \left(x_{i}\right)-b\right)=0\;\; & \forall i=1,\ldots ,n\\ \left(C-\alpha_{1,i}\right)\xi_{1,i}=0\;\; & \forall i=1,\ldots ,n\\ \left(C-\alpha_{2,i}\right)\xi_{2,i}=0\;\; & \forall i=1,\ldots ,n. \end{array}\right.$$

Substituting Equations (5) and (6) into (4), we obtain the following dual form:

(7) $$\begin{array}{cc} \mathrm{maximize}\; & -\frac{1}{2}\sum_{i=1}^{n}\sum_{j=1}^{n}\left(\alpha_{1,i}-\alpha_{2,i}\right)\left(\alpha_{1,j}-\alpha_{2,j}\right)\phi {\left(x_{i}\right)}^{T}\phi \left(x_{j}\right)\\ \; & -\epsilon \sum_{i=1}^{n}\left(\alpha_{1,i}+\alpha_{2,i}\right)\\ \; & +\sum_{i=1}^{n}\left(\alpha_{1,i}-\alpha_{2,i}\right)y_{i}, \end{array}$$

$$\mathrm{subject}\;\mathrm{to}\;\left\{\begin{array}{c} \sum_{i=1}^{n}\left(\alpha_{1,i}-\alpha_{2,i}\right)=0\\ 0\leq \alpha_{1,i}\leq C,\;0\leq \alpha_{2,i}\leq C, \end{array}\right.$$

where $\alpha_{1,i}$ and $\alpha_{2,i}$ denote dual variables (Vapnik [17]). Consequently, the optimization problem in (7) yields the solutions $\hat{w},\hat{b},\hat{f}$ of w,b,f of the following form (Smola and Schölkopf [18]):

$$\hat{w}=\sum_{i=1}^{n}\left(\alpha_{1,i}-\alpha_{2,i}\right)\phi \left(x_{i}\right),$$

$$\hat{b}=\left\{\begin{array}{cc} y_{i}-{\hat{w}}^{T}\phi \left(x_{i}\right)-\epsilon , & 0<\alpha_{1,i}<C\\ y_{i}-{\hat{w}}^{T}\phi \left(x_{i}\right)+\epsilon , & 0<\alpha_{2,i}<C, \end{array}\right.$$

(8) $$\begin{array}{ll} \hat{f}\left(x\right) & =\sum_{i=1}^{n}\left(\alpha_{1,i}-\alpha_{2,i}\right)\phi {\left(x_{i}\right)}^{T}\phi \left(x\right)+\hat{b}\\ & =\sum_{i=1}^{n}\left(\alpha_{1,i}-\alpha_{2,i}\right)K\left(x_{i},x\right)+\hat{b}, \end{array}$$

with $K\left(x,y\right)=\phi {\left(x\right)}^{T}\phi \left(y\right)$. Note that $\hat{w}$ is determined uniquely, whereas $\hat{b}$ is not, although the two cases rarely coincide. One way to determine $\hat{b}$ is to obtain the average of the above two values (Abe [42]).

Furthermore, when constructing a model with the SVR here, we employ the Gaussian kernel for *K* in (8):

$$K\left(x,y\right)=\exp\left(-\frac{||x-{y||}^{2}}{2\gamma^{2}}\right).$$

As such, we have to determine the tuning parameters $\gamma^{2}$, *C* in (3), and ϵ in the loss function (2) appropriately. Accordingly, we consider a cube of $\left(C,\gamma^{2},\epsilon \right)$ with 1≤C≤100, $0.1\leq \gamma^{2}\leq 1$, and 0.1≤ϵ≤1. We then employ a grid search method on this cube.

An analogous approach can be adopted for the GARCH time series (Chen, Härdle and Jeong [13]) by constructing the design matrix X with each row $x_{s}$ comprising the lagged time series of lag *p* and the lagged conditional variance of lag *q*. Specifically, for the GARCH(p,q) model,

(9) $$\begin{array}{cc} x_{s} & ={\left({\overset{˘}{y}}_{s}^{T},{\overset{˘}{\sigma }}_{s}^{T}\right)}^{T},\\ {\overset{˘}{y}}_{s} & ={\left(y_{s-p}^{2},y_{s-p+1}^{2},\ldots ,y_{s-1}^{2}\right)}^{T},\\ {\overset{˘}{\sigma }}_{s} & ={\left(\sigma_{s-q}^{2},\sigma_{s-q+1}^{2},\ldots ,\sigma_{s-1}^{2}\right)}^{T}. \end{array}$$

To reflect the structure above, we present a five-step procedure to obtain the predicted SVR-GARCH function.

Given the time series of length $n+n^{{}^{\prime }}$ and the space of tuning parameters, the procedure is illustrated as follows:

- Step 1. Prescribe the points to be evaluated within this space, then divide the given time series into training and validation time series of size *n* and $n^{{}^{\prime }}$, respectively. This preliminary procedure is required for the subsequent task of validating the fitted SVR-GARCH model, which determines the best tuning parameter sets.
- Step 2. Note that the conditional variance $\sigma_{t}^{2}$ of (9) is unknown. As a remedy, replace $\sigma_{t}^{2}$ with the initial estimates ${\tilde{\sigma }}_{t}^{2}$, which plays the role of a proxy of $\sigma_{t}^{2}$. The estimate ${\tilde{\sigma }}_{t}^{2}$ is based on the training time series using a moving average method (Niemira [43]):
  $${\tilde{\sigma }}_{t}^{2}=\frac{1}{m}\sum_{j=1}^{m}y_{t-j+1}^{2},$$
  where *m* is a positive integer. When *t* is smaller than *m*, ${\tilde{\sigma }}_{t}^{2}$ is computed as an average of the first to the *t*th squares of observations, i.e., ${\tilde{\sigma }}_{t}^{2}=\sum_{j=1}^{t}y_{t-j+1}^{2}/t$ when t<m.
- Step 3. Given a set of tuning parameters, we estimate *g* in (1) with $\hat{g}$ using the SVR with $\sigma_{t}^{2}$ replaced by ${\tilde{\sigma }}_{t}^{2}$. Then, the estimate ${\hat{\sigma }}_{t}^{2}$ of $\sigma_{t}^{2}$ is obtained as:
  $${\hat{\sigma }}_{t}^{2}=\hat{g}\left(y_{t-1}^{2},\ldots ,y_{t-p}^{2},{\tilde{\sigma }}_{t-1}^{2},\ldots ,{\tilde{\sigma }}_{t-q}^{2}\right).$$
- Step 4. Applying the estimated SVR-GARCH model and using the same proxy formula as in Step 2 for the validation time series, the mean absolute error (MAE) is computed as follows:
  $$\mathrm{MAE}=\frac{1}{n^{{}^{\prime }}}\sum_{t=1}^{n^{{}^{\prime }}}\left|{\hat{\sigma }}_{t}^{2}-{\tilde{\sigma }}_{t}^{2}\right|.$$
  The MAE escalates the robustness of the model against outliers and therefore provides more flexibility in a model fitting than the root mean squared error.
- Step 5. Repeat Steps 2 to 4 for all the tuning parameter sets selected in Step 1 and choose the combination that minimizes the MAE. Then, perform Steps 2 and 3 using the training and validation time series together to determine the final model, which is used in obtaining the residuals.

**Remark** **1.** *One might contemplate utilizing different methods regarding the selection of tuning parameters, such as the random search method proposed by Bergstra and Bengio [44]. However, we employ the grid search method in our simulation study because it outperforms the random search method. Furthermore, the choice of m in the construction of proxy volatilities is critical in constructing a stable test. In this study, we report the case of m=5 because it provides reasonably satisfactory results the most consistently.*

**Remark** **2.** *One may consider iteratively updating ${\hat{\sigma }}_{t}^{2}$ until a specific convergent criterion is satisfied, as observed in Chen, Härdle and Jeong [13] and Lee, Lee and Moon [38]. However, the $\hat{g}$ obtained from this iterative approach makes a function either “flatten out” after each iteration, ultimately yielding a constant function, or drastically follow the peak outliers among the initial ${\tilde{\sigma }}_{t}^{2}$’s. Thus, this approach is inadequate and disregarded in our study.*

**Remark** **3.** *Instead of MAE, other loss functions, such as the mean squared error (MSE), the root mean squared error (RMSE), and the mean absolute percentage error (MAPE), could be employed. These loss functions, however, are likely to yield suboptimal results. To illustrate, when either the MSE or the RMSE is used, the result deteriorates as these amplify or shrink the losses according to their values. On the other hand, MAPE yields inconsistent results because it uses unobservable ${\tilde{\sigma }}_{t}^{2}$ rather than ${\hat{\sigma }}_{t}^{2}$.*

## 3. Hybrid CUSUM Test via the SVR-GARCH Model

Based on the work of Lee, Tokutsu and Maekawa [35], Oh and Lee [34] developed the CUSUM of squares test for the GARCH(1,1) model as follows:

$$\begin{array}{cc} y_{t} & =\sigma_{t}\epsilon_{t},\\ \sigma_{t}^{2} & =\omega +\alpha y_{t-1}^{2}+\beta \sigma_{t-1}^{2},\;\;t=1,\ldots ,n, \end{array}$$

where ω>0,α≥0,β≥0,α+β<1, and $\epsilon_{t}$ are i.i.d. random variables with zero mean and unit variance, to perform a test for a parameter change in ω,α,β.

More precisely, the null and alternative hypotheses are formulated as follows:

$$\begin{array}{cc} H_{0} & :\omega ,\alpha ,\beta \;\mathrm{remain}\;\mathrm{the}\;\mathrm{same}\;\mathrm{over}\;t=1,\ldots ,n.\mathrm{vs}.\\ H_{1} & :\mathrm{not}\;H_{0}. \end{array}$$

To test these, ω,α,β are estimated using their consistent estimators $\hat{\omega },\hat{\alpha },\hat{\beta }$, such as Gaussian quasi-maximum likelihood estimates (QMLEs) in Francq and Zakoïan (2004), and the residuals ${\hat{\epsilon }}_{t}$ are recursively obtained via the equation:

$$\begin{array}{ccc} {\hat{\epsilon }}_{t} & = & \frac{y_{t}}{{\hat{\sigma }}_{t}},\\ {\hat{\sigma }}_{t}^{2} & = & \hat{\omega }+\hat{\alpha }y_{t-1}^{2}+\hat{\beta }\sigma_{t-1}^{2},\;\;t=1,\ldots ,n, \end{array}$$

with the initials $y_{0}=0,\;\sigma_{0}^{2}=y_{1}$. Subsequently, the CUSUM of squares test is given by

$$\begin{array}{c} {\hat{T}}_{n}=\max_{1\leq k\leq n}\frac{1}{\sqrt{n}{\hat{\tau }}_{n}}|\sum_{t=1}^{k}{\hat{\epsilon }}_{t}^{2}-\frac{k}{n}\sum_{t=1}^{n}{\hat{\epsilon }}_{t}^{2}| \end{array}$$

with

$$\begin{array}{c} {\hat{\tau }}_{n}^{2}=\frac{1}{n}\sum_{t=1}^{n}{\hat{\epsilon }}_{t}^{4}-{\left(\frac{1}{n}\sum_{t=1}^{n}{\hat{\epsilon }}_{t}^{2}\right)}^{2}. \end{array}$$

Letting

$$\begin{array}{c} T_{n}=\max_{1\leq k\leq n}\frac{1}{\sqrt{n}\tau }|\sum_{t=1}^{k}\epsilon_{t}^{2}-\frac{k}{n}\sum_{t=1}^{n}\epsilon_{t}^{2}|, \end{array}$$

with $\tau^{2}=Var\left(\epsilon_{1}^{2}\right)$, Oh and Lee [34] verified that under regularity conditions, ${\hat{T}}_{n}$ behaves asymptotically the same as $T_{n}$ under $H_{0}$ as *n* tends to ∞, and thus, the limiting null distribution of ${\hat{T}}_{n}$ is the same as $\sup_{0\leq s\leq 1}\left|B^{\circ }\left(s\right)\right|$, where $B^{\circ }$ denotes a Brownian bridge on the unit interval because $T_{n}$ converges to the supremum of a Brownian bridge in distribution due to Donsker’s invariance principle; see Billingsley [45]. The critical values then can be obtained asymptotically. For instance, the null hypothesis $H_{0}$ of no changes is rejected if ${\hat{T}}_{n}\geq 1.3397$ at the level of 0.05, which can be obtained through Monte Carlo simulations. Furthermore, provided that a change point exists, the location of change is identified as:

$${\hat{k}}_{n}={\mathrm{argmax}}_{1\leq k\leq n}|\sum_{t=1}^{k}{\hat{\epsilon }}_{t}^{2}-\frac{k}{n}\sum_{t=1}^{n}{\hat{\epsilon }}_{t}^{2}|.$$

Multiple change points can be detected by following the scheme of Inclán and Tiao [24]. The same approach can be adopted for more general nonlinear location-scale time series models as seen in Oh and Lee [32].

This CUSUM procedure can be applied to the residuals obtained through the SVR-GARCH models, as can be seen in our simulation study, since they will quite likely behave like the true errors $\epsilon_{t}$. Lee, Lee and Moon [38] recently adopted the SVR-ARMA scheme to calculate the residuals in the change point detection problem on ARMA models and affirmed its validity.

In the next section, we evaluate the SVR-GARCH model in the previous section for various settings, wherein we only consider the case of p=q=1 as most financial time series can be sufficiently described with GARCH(1,1) models (Hansen and Lunde [46]).

## 4. Simulation Results

In this section, we evaluate the performance of the SVR-GARCH model through simulations using the linear, asymmetric, threshold, GJR, and exponential GARCH models. For this task, we generate a time series of length n= 500, 1000, and 2000 to evaluate the empirical sizes and powers at the level of 0.05. The experiment is comprised of the following four steps.

- Step 1. Generate a time series of length 2n from a prescribed GARCH model.
- Step 2. Follow the estimation scheme described in Section 3 with m=5. In this procedure, the first 0.7*n* number of time series constitute the training set, and the following 0.3n number of time series constitute the validation set.
- Step 3. Conduct the CUSUM of squares test described in Section 3. We utilize the remaining *n* number of time series as a testing set.
- Step 4. Repeat Steps 1 to 3 1000 times iteratively, and then, compute the empirical sizes and powers.

In the power evaluation, we assume that the change point existed in the middle of the time series. Here, we utilize R 3.5.1 running on Windows 10 and the packages “e1071” and “fGarch”.

For this task, various GARCH models are considered as listed below, wherein $\epsilon_{t},t=1,\ldots ,2n$, denote i.i.d. normal errors with zero mean and unit variance:

- GARCH(1,1) model:
  $$\begin{array}{cc} y_{t} & =\sigma_{t}\epsilon_{t},\\ \sigma_{t}^{2} & =\omega +\alpha y_{t-1}^{2}+\beta \sigma_{t-1}^{2},\\ \alpha & \geq 0,\beta \geq 0. \end{array}$$
- AGARCH(1,1) model:
  $$\begin{array}{cc} y_{t} & =\sigma_{t}\epsilon_{t},\\ \sigma_{t}^{2} & =\omega +\alpha {\left(y_{t-1}-b\right)}^{2}+\beta \sigma_{t-1}^{2},\\ \alpha & \geq 0,\beta \geq 0. \end{array}$$
- GJR-GARCH(1,1) model:
  $$\begin{array}{cc} y_{t} & =\sigma_{t}\epsilon_{t},\\ \sigma_{t}^{2} & =\omega +\alpha_{1}{y_{t-1}^{+}}^{2}+\alpha_{2}{y_{t-1}^{-}}^{2}+\beta \sigma_{t-1}^{2},\\ y_{t}^{+} & =\max\left(y_{t},\;0\right),\;y_{t}^{-}=-\min\left(y_{t},\;0\right),\\ \alpha_{1} & \geq 0,\alpha_{2}\geq 0,\beta \geq 0. \end{array}$$
- TGARCH(1,1) model:
  $$\begin{array}{cc} y_{t} & =\sigma_{t}\epsilon_{t},\\ \sigma_{t} & =\omega +\alpha |y_{t-1}|+\beta \sigma_{t-1},\\ \alpha & \geq 0,\beta \geq 0. \end{array}$$
- Log-linear GARCH(1,1) model (a specific variation of the EGARCH(p,q) model):
  $$\begin{array}{cc} y_{t} & =\sigma_{t}\epsilon_{t},\\ \log\sigma_{t}^{2} & =\omega +\alpha \log y_{t-1}^{2}+\beta \log\sigma_{t-1}^{2}. \end{array}$$

When examining empirical power, we first consider the change in parameters. To illustrate this, we consider the change in ω, the sum of α and β for the GARCH, TGARCH, and log-linear GARCH models, and the change in *b* for the AGARCH model. Similarly, for the GJR-GARCH model, we consider a change in the sum of $\alpha_{1}$, $\alpha_{2}$, and β instead. Under the null hypothesis, the parameters are set as follows:

1. GARCH model: ω=0.3,α=0.3,β=0.3;
2. AGARCH model: ω=0.3,α=0.3,β=0.4,b=1;
3. GJR-GARCH model: $\omega =0.3,\;\alpha_{1}=0.3,\;\alpha_{2}=0.4,\;\beta =0.3$;
4. TGARCH model: ω=0.3,α=0.3,β=0.3;
5. log-linear GARCH model: ω=0.3,α=0.3,β=0.3.

Table 1 and Table 2 indicate the results for the GARCH and AGARCH models, respectively. In both models, no size distortions are observed in most cases. Furthermore, the results confirm that the test performs well in general, in terms of power. Furthermore, as anticipated, the power increases as the sample size increases. Table 3, Table 4 and Table 5 depict the results for the GJR-GARCH, TGARCH, and log-linear GARCH models, respectively. Although some mild distortions of size can be noticed in the GJR-GARCH model, the results are mostly similar to those of the GARCH and AGARCH models. For the log-linear GARCH model, the truncated residuals are employed in the construction of the CUSUM test, that is,

$${\tilde{\epsilon }}_{t}:={\hat{\epsilon }}_{t}I(|{\hat{\epsilon }}_{t}\left|<N\right)+NI(|{\hat{\epsilon }}_{t}\left|\geq N\right)$$

with N=20 to mitigate the influence of extreme outliers. This modification does not hamper the asymptotic behavior of the CUSUM of squares test, whereas it significantly improves the performance of the CUSUM test in terms of stability and power, as demonstrated in Table 5.

Finally, to simulate the situation in which the underlying model is unknown, we examine the case where both the parameters and the underlying model itself change. Analogously to the simulation study regarding the above log-linear GARCH model, we truncate the residuals and implement ${\tilde{\epsilon }}_{t}$ to formulate the CUSUM statistic in Cases 1 and 2:

1. GARCH(1,1) changes to log-linear GARCH(1,1);
2. log-linear GARCH(1,1) changes to GARCH(1,1);
3. TGARCH(1,1) changes to AGARCH(1,1);
4. AGARCH(1,1) changes to GJR-GARCH(1,1).

Table 6 shows that the test produces powers comparable to those with only parameter changes. Overall, our findings strongly support the validity of the SVR-GARCH model for detecting a change.

## 5. Real Data Analysis

**Figure 1:**
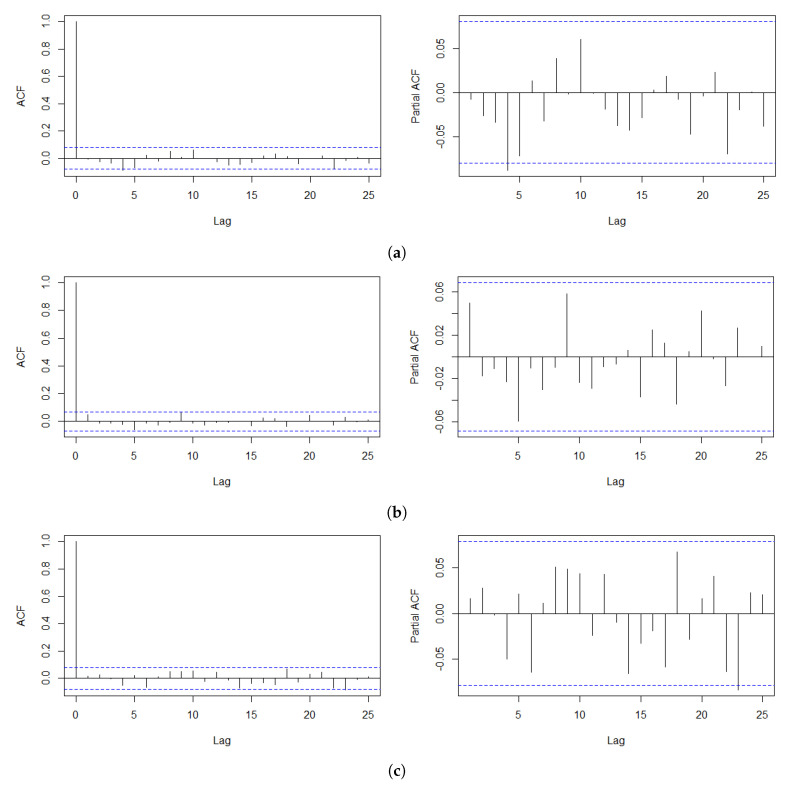
Plot of ACF and partial autocorrelation function (PACF) up to lag 25 of log-returns of the (**a**) S&P 500, (**b**) KOSPI, and (**c**) KRW/USD indices.

**Figure 2:**
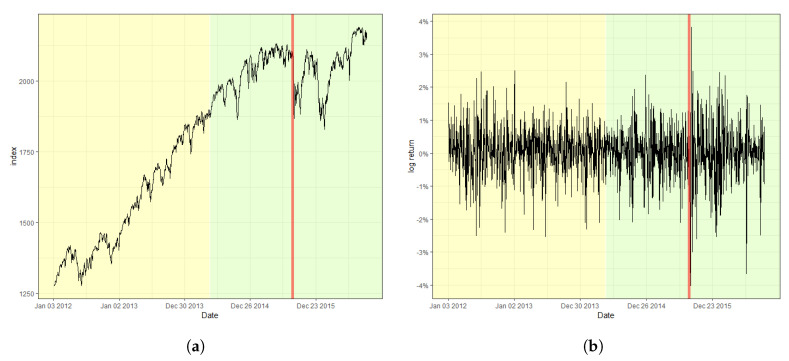
Plot of (**a**) the raw index of S&P 500 and (**b**) its log-returns with the detected change point.

**Figure 3:**
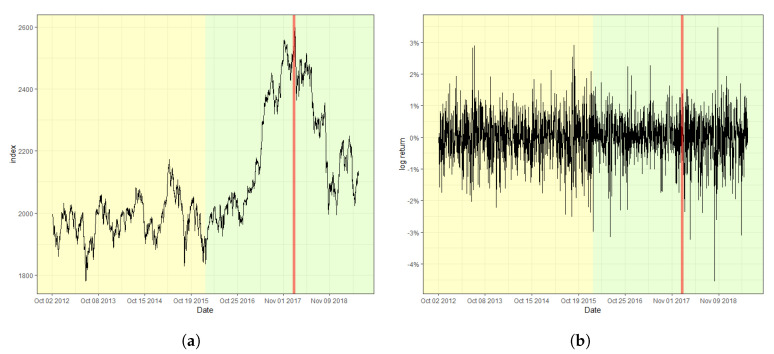
Plot of (**a**) the raw index of KOSPI and (**b**) its log-returns with the detected change point.

**Figure 4:**
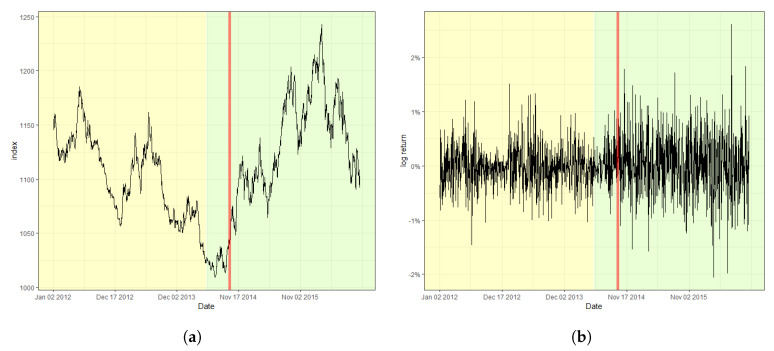
Plot of (**a**) the KRW/USD index and (**b**) its log-returns with the detected change point.

In this section, we analyze the log-returns of the daily index of the S&P 500 from 3 January 2012 to 30 September 2016, the Korea Composite Stock Price Index (KOSPI) from 2 October 2012 to 28 June 2019, and the exchange rate between U.S. dollars (USD) and South Korean Won (KRW), denoted as KRW/USD, from 2 January 2012 to 30 September 2016. We obtain these time series from the website “investing.com”. The two South Korean economic indices are selected because they are well appreciated to be susceptible to various international affairs of the country owing to its geographical location and heavy dependence on the exports of the South Korean economy. Prior to fitting the SVR-GARCH model, we first inspect the autocorrelation function (ACF) and partial autocorrelation function (PACF) of the log-returns of each training time series, consisting of the first half of the entire time series, to examine the presence of irregular patterns of autocorrelations. This step is needed to verify the adequacy of the training time series. Figure 1 shows that the ACFs and PACFs for all three training datasets support stationarity to a great extent, indicating that estimation via the SVR-GARCH model with these training time series would not undermine the outcomes. The selected training datasets are highlighted with yellow shaded areas in Figure 2, Figure 3 and Figure 4. For example, the training period of the S&P 500 index is from 2 April2012 to 16 April 2014.

Figure 2 displays the S&P 500 index and its log-returns with the detected change point, 18 August 2015, indicated by a red vertical line. The obtained value of the CUSUM statistic in this case is 2.4751, which rejects the null hypothesis of a lack of changes at the nominal level of 0.05. The result signifies that the index fluctuates more heavily after the change point in comparison with that before the change point. Moreover, (a) of Figure 2, the original index, shows a relatively consistent trend of steady increase before the change point. However, a double-dip is observed after the change point, which coincides with the fact that a change in trend is often accompanied by a change in variation in stock markets. Furthermore, Figure 3 and Figure 4 plot the result of the change point detection process for the KOSPI and KRW/USD indices, respectively. Here, we obtain the CUSUM statistic values of 1.5081 and 4.2754, respectively, which indicates the detection of a change in both cases at the level of 0.05. The identified location of a change point for the KOSPI index appears to be 22 January 2018, whereas that of KRW/USD is 26 September 2014. Similar to the case of the S&P 500 index, a significant change in trend is also observed in the original datasets, as shown in the plot (a) of Figure 3 and Figure 4. Our finding also illuminates that the log-return of the KRW/USD index experiences a more significant change in volatility, in contrast to the other two cases.

In general, it is not feasible to find the matching incidents that cause change points in economic indices either because they can be obscured from media or they may affect the market with a significant delay. Nevertheless, for the KOSPI example, we could deduce that unstable international affairs could be the culprit behind such changes. These affairs include the nuclear weapon test of North Korea in September 2017, and the participation of North Korea in the Pyeongchang Winter Olympic Games in February 2018, which caused massive turmoil in the Korean peninsula. However, one could argue that the raising of the interest rate by the FRB twice in March and June 2018, was a much more significant factor affecting both the changes in volatility and trend, considering that the impact of the international affairs of the Korean peninsula has been limited on many occasions. In contrast, in the case of the KRW/USD index, the report from the Bank of Korea in January 2015, reasoned that the high volatility after the change point was due to a byproduct of ending quantitative easing and the accompanying improvement of the U.S. economy.

## 6. Conclusions

In this study, we proposed the CUSUM of squares test based on the residuals obtained with the SVR-GARCH model in order to detect a parameter change in the volatility of time series. Monte Carlo simulations were conducted with various linear and nonlinear GARCH models, including the GARCH, GJR-GARCH, AGARCH, TGARCH, and log-linear GARCH models, and the obtained results confirmed the validity of the SVR-GARCH method. Our method was then applied to the analysis of financial datasets such as the S&P 500, KOSPI, and KRW/USD indices and detected one change in all cases. Overall, our findings supported the validity of our method and the practicality in financial time series analysis. Here, we only considered a plain SVR method for emphasizing the hybrid of the CUSUM and SVR methods and for easy access to general readers. However, more sophisticated methods could be employed for refinement concerning the selection of tuning parameters and kernel functions, although they do not necessarily guarantee a better performance, possibly due to over-fitting; see Remark 2 of Lee, Lee and Moon [38]. Furthermore, in this study, we only investigated the retrospective change point test. However, a similar method could be applied to an on-line monitoring process (Huh, Oh and Lee [47]), which aims at an early detection of an anomaly in sequentially observed time series. In this case, the training data could be redesigned via a rolling window procedure. As these issues go beyond the scope of the current study, we leave them to our future project.

## Figures and Tables

**Table 1:** Empirical sizes and powers for the GARCH(1,1) model.

ω=0.3				
α=0.3		n=500	n=1000	n=2000
β=0.3				
size		0.023	0.038	0.055
change of ω	→ω=1	0.761	0.826	0.956
	→ω=0.1	0.612	0.792	0.97
change of α,β	→α=0.1,β=0.2	0.355	0.651	0.949
	→α=0.4,β=0.5	0.649	0.802	0.952
change of mixed parameters	→ω=0.7,α=0.1	0.871	0.969	0.981
	→ω=0.1,β=0.1	0.848	0.952	0.964

**Table 2:** Empirical sizes and powers for the AGARCH(1,1) model.

ω=0.3				
α=0.3,β=0.4		n=500	n=1000	n=2000
b=1				
size		0.031	0.04	0.03
change of ω	→ω=1	0.881	0.951	0.975
	→ω=0.1	0.26	0.613	0.904
change of α,β	→α=0.1,β=0.1	0.783	0.939	0.975
	→α=0.6	0.762	0.863	0.941
change of *b*	→b=0	0.591	0.897	0.976
	→b=3	0.898	0.936	0.957
change of mixed parameters	→ω=0.1,α=0.5,β=0.4,b=0	0.565	0.726	0.846
	→ω=0.1,β=0.6,b=2	0.879	0.926	0.966

**Table 3:** Empirical sizes and powers for the GJR-GARCH(1,1) model.

ω=0.3				
$\alpha_{1}=0.3,\;\alpha_{2}=0.4$		n=500	n=1000	n=2000
β=0.3				
size		0.021	0.029	0.028
change of ω	→ω=1	0.851	0.912	0.947
	→ω=0.1	0.644	0.787	0.866
change of $\alpha_{i},\;\beta \left(i=1,2\right)$	$\to \;\alpha_{1}=0.5,\;\alpha_{2}=0.8$	0.418	0.695	0.879
	$\to \;\alpha_{2}=0.1,\;\beta =0.1$	0.421	0.751	0.888
change of mixed parameters	$\to \;\omega =0.5,\;\alpha_{2}=0.6$	0.657	0.863	0.928
	→ω=0.1,β=0.7	0.717	0.857	0.925

**Table 4:** Empirical sizes and powers for the TGARCH(1,1) model.

ω=0.3				
α=0.3		n=500	n=1000	n=2000
β=0.3				
size		0.037	0.049	0.059
change of ω	→ω=1	0.718	0.795	0.879
	→ω=0.1	0.647	0.84	0.902
change of α,β	→α=0.1,β=0.2	0.805	0.886	0.913
	→α=0.4,β=0.5	0.735	0.838	0.897
change of mixed parameters	→ω=0.7,α=0.1	0.907	0.97	0.994
	→ω=0.1,β=0.1	0.499	0.674	0.772

**Table 5:** Empirical sizes and powers for the log-linear GARCH(1,1) model.

ω=0.3				
α=0.3		n=500	n=1000	n=2000
β=0.3				
size		0.047	0.039	0.037
change of ω	→ω=1	0.906	0.984	0.997
	→ω=0.1	0.228	0.382	0.507
change of α,β	→α=−0.1,β=−0.2	0.868	0.946	0.976
	→α=−0.4,β=0.5	0.917	0.985	1
change of mixed parameters	→ω=0.7,β=0.5	0.82	0.973	0.998
	→ω=0.1,α=−0.1	0.862	0.944	0.971

**Table 6:** Empirical powers for model changes (log-linear GARCH = log-GARCH).

ω=0.3				
**α=0.3**		**n=500**	**n=1000**	**n=2000**
β=0.3				
GARCH → log-GARCH	→ω=1	0.879	0.946	0.956
	→α=0.1,β=0.1	0.668	0.918	0.969
log-GARCH → GARCH	→ω=1	0.907	0.97	0.994
	→α=0.1,β=0.1	0.499	0.674	0.772
TGARCH → AGARCH	→ω=1,b=1	0.728	0.795	0.879
	→α=0.1,β=0.1,b=1	0.851	0.891	0.919
ω=0.3				
α=0.3,β=0.3		**n=500**	**n=1000**	**n=2000**
b=1				
AGARCH → GJR-GARCH	$\to \;\omega =0.7,\;\alpha_{1}=0.3,\;\alpha_{2}=0.7,\;b=0$	0.549	0.861	0.958
	$\to \;\alpha_{1}=0.1,\;\alpha_{2}=0.3,\;\beta =0.1,\;b=0$	0.802	0.931	0.976

## Abbreviations

The following abbreviations are used in this manuscript:

CUSUM	cumulative sum
SVR	support vector regression
SVM	support vector machine
GARCH	generalized autoregressive conditionally heteroscedastic
EGARCH	exponential GARCH
GJR-GARCH	Glosten, Jagannathan, and Runkle-GARCH
TGARCH	threshold GARCH
APARCH	asymmetric power ARCH
NN	neural network
ARMA	autoregressive and moving average
QMLE	quasi-maximum likelihood estimator
KOSPI	Korea Composite Stock Price Index
KRW	Korean Won
VaR	value at risk
ES	expected shortfall
